# A liquid biopsy to detect multidrug resistance and disease burden in multiple myeloma

**DOI:** 10.1038/s41408-020-0304-7

**Published:** 2020-03-13

**Authors:** Sabna Rajeev Krishnan, Gabriele De Rubis, Hayley Suen, Douglas Joshua, Yiu Lam Kwan, Mary Bebawy

**Affiliations:** 1 Graduate School of Health, Discipline of Pharmacy, University of Technology Sydney, Ultimo, NSW 2007 Australia; 2 Institute of Haematology, Royal Prince Alfred Hospital, Camperdown, NSW 2050 Australia; 3 Department of Haematology, Concord Repatriation General Hospital, Concord, NSW 2139 Australia

**Keywords:** Myeloma, Cancer therapeutic resistance, Myeloma, Myeloma, Cancer stem cells

## Abstract

Multiple myeloma is an incurable cancer of bone marrow plasma cells, with a 5-year survival rate of 43%. Its incidence has increased by 126% since 1990. Treatment typically involves high-dose combination chemotherapy, but therapeutic response and patient survival are unpredictable and highly variable—attributed largely to the development of multidrug resistance (MDR). MDR is the simultaneous cross-resistance to a range of unrelated chemotherapeutic agents and is associated with poor prognosis and survival. Currently, no clinical procedures allow for a direct, continuous monitoring of MDR. We identified circulating large extracellular vesicles (specifically microparticles (MPs)) that can be used to monitor disease burden, disease progression and development of MDR in myeloma. These MPs differ phenotypically in the expression of four protein biomarkers: a plasma-cell marker (CD138), the MDR protein, P-glycoprotein (P-gp), the stem-cell marker (CD34); and phosphatidylserine (PS), an MP marker and mediator of cancer spread. Elevated levels of P-gp^**+**^ and PS^**+**^ MPs correlate with disease progression and treatment unresponsiveness. Furthermore, P-gp, PS and CD34 are predominantly expressed in CD138^−^ MPs in advanced disease. In particular, a dual-positive (CD138^**−**^P-gp^**+**^CD34^**+**^) population is elevated in aggressive/unresponsive disease. Our test provides a personalised liquid biopsy with potential to address the unmet clinical need of monitoring MDR and treatment failure in myeloma.

## Introduction

Multiple myeloma (MM) is a neoplasia of terminally differentiated plasma cells, characterised by the presence of multiple bone marrow infiltrates^1^. It represents the second most commonly diagnosed haematological malignancy worldwide^2^, with 159,985 cases reported globally in 2018^3^. First-line treatment includes high-dose combination chemotherapy with or without autologous stem-cell transplant (ASCT). The presence of multisite tumour infiltrates, each with differing degrees of drug sensitivity^4^, contribute to a tumour heterogeneity and variability in survival, ranging from a few weeks to more than 10 years^5–7^. MM is also marked by multiple recurrent episodes of remission and relapse, the latter being a clinical manifestation of disease unresponsiveness to treatment and resistance to chemotherapy^8^.

Multidrug resistance (MDR) is a unique type of resistance in which cancer cells become cross-resistant to a wide range of structurally and functionally unrelated drugs usually following exposure to a single chemotherapeutic agent^9^. One of the most important mechanisms by which cancers acquire MDR is through the overexpression of resistance proteins, belonging to the ATP class of drug transporters, including P-glycoprotein (P-gp)^10^. These are plasma membrane drug efflux transporters that mediate the removal of chemotherapeutics from the cancer cell plasma membrane^10^. Elevated P-gp expression is correlated with poor prognosis and response to chemotherapy across many cancers^9^. In the context of MM, P-gp expression increases by up to 75% in patients following treatment^11^. The utility of newer immunomodulatory drugs and proteosome inhibitors are also compromised, with evidence that they are also P-gp substrates^12–14^. Likewise, many of the agents typically used in combination chemotherapy for MM are also P-gp substrates. Currently, there is no procedure that supports a minimally invasive and continuous monitoring for the presence and development of MDR in MM during treatment.

Microparticles (MPs) are a subset of extracellular vesicles (0.1−1 µm in diameter) released from the plasma membranes of most cell types^15^. MPs differ from other extracellular vesicles by virtue of their size, biogenesis and cargo^16–18^. Circulating MPs have been detected systemically for many cancers including MM, breast, prostate, and lung cancer^19–24^. Their presence in blood make them important components of the ‘tumour circulome’ and ideal candidates as biomarkers in the context of a liquid biopsy^25^.

We discovered that cancer-derived MPs confer the transfer and spread of MDR within cancer cell populations^26–30^ through the intercellular transfer of functional resistance proteins and nucleic acids packaged within the vesicular cargo^26,27,30^. Circulating MPs are hence promising surrogate markers of compartmentally confined malignancies such as MM.

In our previous work, we demonstrated the clinical feasibility of analysing circulating MPs in MM, whereby the number of CD138^+^ circulating MPs was elevated in MM patients across all stages of disease and corresponds to plasma-cell burden and treatment response in individual patients^23^.

We now expand on our initial findings and present a novel blood test with capacity to continuously monitor patients for the presence of MDR during treatment. This minimally invasive blood test accounts for the presence of multisite tumour infiltrates, can test for the presence of MDR during routine follow-up and allows for simultaneous analysis of tumour burden. The technology complements existing gold standard tests, has potential to support clinical staging criteria and streamlines easily into existing hospital workflows.

## Materials and methods

### Reagents and antibodies

Annexin V-V450, anti-CD138-APC, anti-CD41a-PE, anti-P-gp-FITC, anti-CD34-PE*-*Cy7, matched isotype controls, BD^TM^ CompBeads anti-mouse-Ig k, Sphero^TM^ Rainbow calibration particles and TruCount^TM^ tubes were from BD Biosciences (Sydney, NSW, Australia). Latex beads of 0.3 and 1.1 µm diameter were from Sigma-Aldrich (Sydney, NSW, Australia). Details of other reagents used are described in Supplementary Table 1.

### Study design and eligibility criteria

This study was approved by the Sydney Local Health District Human Research Ethics Committee (HREC) of Concord Repatriation General Hospital (CRGH) (HREC/11/CRGH/223-CH62/6/2011-150), Royal Prince Alfred Hospital (RPAH) HREC (SSA/12/RPAH/10) and the University of Technology Sydney (2012-004R). Blood samples were collected from myeloma patients and healthy subjects (>18 years of age) after informed consent at the CRGH and RPAH blood collection centres in accordance with the Declaration of Helsinki. A predetermined sample size was not calculated as, being a preliminary study, we analysed all available samples following informed consent. Study participants were de-identified and assigned a code for access to clinical information. Healthy subjects were age-matched, non-cancer patients with normal haematology and devoid of any cytotoxic treatment or radiotherapy of any nature during the previous 5 years. Pregnancy was an exclusion criterion. In total, we assessed three markers (P-gp, CD138, PS) independently in 74 patients. Of the myeloma cohort this included patients that were treatment responsive (partial remission (*n* = 30) and complete remission (*n* = 12)), de novo (*n* = 14) and relapsed (*n* = 18). We also assessed the complete signature comprising four markers (P-gp, CD138, PS, CD34) in 11 patients. Patient clinical response was established according to the IMWG guidelines^31,32^.

### Sampling, MP enrichment and immunolabelling

Following informed consent, 8 ml of blood was taken in Ethylenediaminetetraacetic acid (EDTA) tubes from each patient and healthy donors. The samples were assigned a code with date and time of collection.

Platelet-free plasma (PFP) was prepared as previously described^33,34^. Briefly, whole blood was centrifuged immediately after sampling at 1500 × *g* for 20 min at room temperature to remove cells, and the platelet-poor plasma obtained was subsequently centrifuged at 13,000 × *g* for 2 min at room temperature to obtain PFP. The PFP was divided into 200 µl aliquots and centrifuged at 18,890 × *g*, 4 °C for 30 min to pellet the MP fraction. The samples were analysed immediately after isolation or kept at −20 °C until analysis. Prior to immunolabelling, frozen samples were thawed on ice. Samples were resuspended in 500 µl cold PBS and spun at 18,890 × *g*, 30 min, 4 °C. After removing the supernatant, the MP pellet was immunolabelled by re-suspending the pellet with 20 µl of anti-CD41a-PE antibody, 20 µl of anti-P-gp-FITC antibody, 5 µl of anti-CD138-APC and 5 µl of CD34-PE-Cy7 antibody for 30 min in the dark at room temperature. The same volumes and labelling conditions were performed for the respective isotype-matched control antibodies also. Following labelling, the pellet was washed with 500 µl ice-cold PBS and centrifuged at 18,890 × g for 30 min at 4 °C. The pellet was subsequently resuspended in 5 µl Annexin V-V450 and 5 µl Annexin Binding Buffer 10× and incubated for 20 min at room temperature in the dark. The sample was then diluted by the addition of 500 µl of Annexin V Binding Buffer 1×. Isotype-matched control samples were resuspended in 500 µl PBS after staining. The samples were transferred to BD Trucount™ Tubes (BD Biosciences, Australia) for flow cytometric analysis.

### Flow cytometric analysis

The phenotyping and quantitatation of MPs in patient samples were performed using a Becton−Dickinson LSRII and a Becton−Dickinson LSRFortessa X20 flow cytometer. Technical specifications and laser and filter configurations for the channels used are outlined in Supplementary Tables 2, 3. Flow cytometer setup was performed using CS&T instrument setup beads (BD Australia, North Ryde, NSW) following the manufacturer’s instructions. Triggering thresholds as well as FSC and SSC voltages were set to exclude signal from PBS alone. An MP size gate was defined on a FSC-A vs. SSC-A dotplot by using 0.3 and 1.1 µm latex beads (Sigma-Aldrich, Australia) as the lower and upper size limits, respectively^23^. Compensation setup was performed using CompBeads compensation particles (BD Australia, North Ryde, NSW), following the manufacturer’s instructions. Compensation matrices were calculated and applied using the setup feature in the BD FACSDiva Software (Version 8.0.1). Laser performance was validated prior to each experiment using Sphero Rainbow calibration particles (BD Australia, North Ryde, NSW). A sequential gating strategy using the MP size gate followed by gating for CD41a^−^ events (to exclude platelet-derived MPs) and CD138^**+**^ was applied to MP populations. Further, CD138^+/−^ subpopulations were gated based on staining for Pgp, CD34 and PS. The full gating strategy is shown in Supplementary Fig. 1. Relevant isotype-matched and unstained controls were included in the analysis. Total MP counts comprised all CD41a^−^ events in the MP size gate. Samples were run at the lowest possible sample pressure and flow rate (~8–12 µl/min) in order to minimise the occurrence of swarm detection and coincidence. Sample acquisition was performed using the collection of 2000−10,000 Trucount™ beads events as a stopping parameter.

Fluorescence positivity gates were set based on the background fluorescence observed in isotype-matched control or unstained controls. Data analysis was performed using the BD FACSDiva and CellQuest Pro softwares. MP Numbers (MPs/µl) were calculated according to the Trucount™ tubes’ manufacturer’s formula: MPs/µl = [#MPs region of interest/Trucount™ beads analysed] × [Trucount™ beads per tube/Vol. sample added to Trucount™ tube].

### Statistical analysis

Normality of data distribution for each group and each parameter was assessed by performing a Shapiro−Wilk test. The Mann–Whitney (*U*) test was performed for the non-parametric data obtained. The software used for statistical analysis was GraphPad Prism® version 7.0 for Mac (GraphPad, La Jolla, CA, USA). Mann–Whitney constant *U* and *p* value are also indicated. The results with a two-tailed *p* value, *p* < 0.01(**) and *p* < 0.05 (*), were considered significant.

## Results

### P-gp^+^ MPs are present in plasma of myeloma patients

**Fig. 1:**
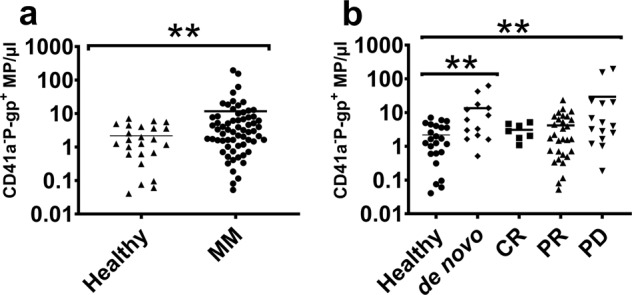
P-gp^+^ MPs increase in MM. The P-gp^**+**^ MP counts in the total MP population in MM patients and healthy subjects are shown. **a** P-gp^**+**^ MP numbers were significantly greater in MM patients relative to healthy subjects (*p* = 0.0071, *U* = 516). **b** P-gp^**+**^ MP numbers were greater in de novo (*p* = 0.0032, *U* = 69) and progressive disease (*p* = 0.0096, *U* = 96) patients relative to healthy subjects. There was no significant difference in the P-gp^**+**^ MP numbers between patients in partial remission (PR) or complete remission (CR) and healthy subjects. Lines represent the mean values. *p* values were determined by Mann–Whitney *U*. **p < 0.01.

MPs isolated from the plasma of myeloma patients and healthy volunteers were immunophenotyped for the presence of P-gp. This is the first demonstration of the presence of the MDR protein P-gp on the surface of circulating MPs in myeloma or in any other cancer. We observed a 5.4-fold greater number of P-gp^+^ MPs in myeloma patients relative to healthy subjects (Fig. 1a). More specifically, P-gp^**+**^ MPs were 6.2- and 13.5-fold higher in de novo and progressive disease (PD) patients, respectively, compared to healthy subjects (Fig. 1b). There was no significant difference in P-gp^**+**^ MP numbers between patients in complete remission (CR) and partial remission (PR) relative to healthy subjects (Fig. 1b).

### CD138 and P-gp do not coexpress on MPs in myeloma Patients

**Fig. 2:**
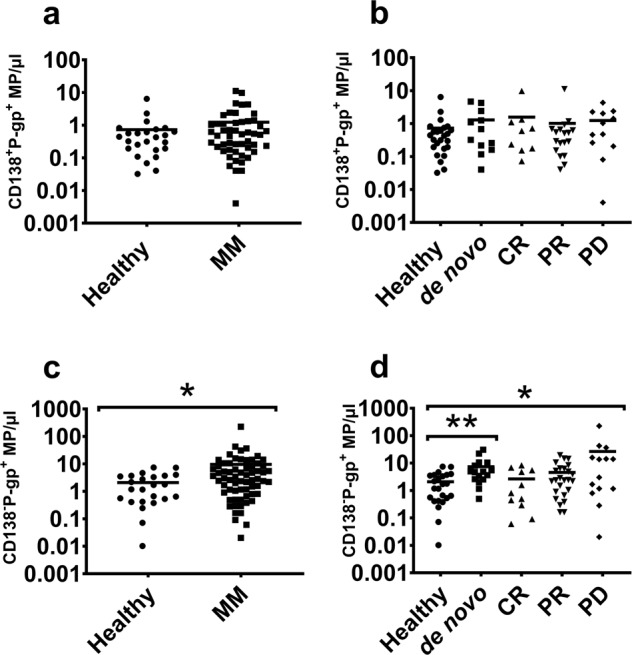
CD138 and P-gp are not coexpressed on MPs. The numbers of Pgp^+^ MPs in the context of CD138 expression are shown. **a** There was no significant difference in CD138^**+**^ P-gp^**+**^ MP numbers in MM patients relative to healthy subjects. **b** CD138^**+**^ P-gp^**+**^ MP numbers across de novo, PR, CR and PD were not significantly different in MM patients relative to healthy subjects. **c** CD138^**−**^ P-gp^**+**^ MP numbers were significantly greater in MM patients relative to healthy subjects (*p* = 0.011, *U* = 541). **d** CD138^**−**^ P-gp^+^ numbers were significantly greater in de novo (*p* = 0.001, *U* = 81) and PD (*p* = 0.0379, *U* = 104) patients compared to healthy volunteers and not significant for CR, PR patients. Lines represent the mean values. *p* values were determined by Mann–Whitney *U*. *p < 0.05; **p < 0.01.

We observed no significant difference in CD138^**+**^ P-gp^**+**^ MPs between myeloma patients and healthy subjects (Fig. 2a). We also observed no significant difference across de novo, CR, PR and PD states relative to healthy subjects (Fig. 2b). Conversely, we observed a significant 4.6-fold increase in CD138^**−**^ P-gp^**+**^ MPs in myeloma patients relative to healthy subjects (Fig. 2c). The CD138^**−**^ P-gp^**+**^ MP counts were 3.5 and 12.67-fold greater in de novo and PD patients, respectively, compared to healthy subjects. CD138^**−**^ P-gp^**+**^ MP numbers were not significantly different in the CR or PR patient subsets relative to healthy subjects (Fig. 2d).

### An increase in PS^+^ MPs is associated with progressive disease

PS is a phospholipid that is preferentially exposed at high levels on the exoplasmic surface of cancer and metastatic cell plasma membranes, and which is gaining importance as a cancer cell targeting biomarker^35^. Increased PS has been correlated with tumour aggressivity^36^ and its presence on MPs has been shown to be associated with neovascularization through interactions with vascular endothelial cells^37^. PS is also an MP marker, its presence arising from the loss of phospholipid asymmetry during MP biogenesis^18^. PS^+^ MP counts hence also coincide with increased tumour burden.

**Fig. 3:**
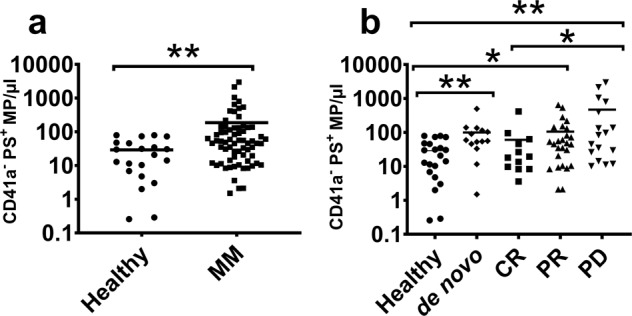
PS^+^ MPs represents a more aggressive state in MM. The PS^**+**^ MP counts in MM patients and healthy subjects are shown. **a** PS^**+**^ MP counts were significantly greater in MM patients relative to healthy volunteers (*p* = 0.005, *U* = 433). **b** PS^**+**^ MP counts were greater in de novo (*p* = 0.0026, *U* = 60), PR (*p* = 0.03, *U* = 172), and PD (*p* = 0.005, *U* = 85) cohorts relative to healthy volunteers. No significant difference in PS^**+**^ MP counts was observed between the CR and healthy volunteers. PS^+^ MPs counts were significantly higher in the PD cohort compared to the CR cohort (*p* = 0.034, *U* = 54). *p* values were generated using Mann–Whitney *U* test and the lines represent mean values. *p < 0.05; **p < 0.01.

Total PS^**+**^ MPs were significantly (6.4-fold) greater in myeloma patients relative to healthy subjects (Fig. 3a). In particular, PS^**+**^ MP counts were 3.4- and 3.6-fold greater in the de novo and PR cohorts respectively, relative to healthy volunteers (Fig. 3b). Most importantly, the PS^**+**^ MP counts were 16-fold higher in the PD cohort relative to healthy subjects. We did not observe any significant difference in counts between CR and healthy subjects (Fig. 3b). The PS^+^ MPs counts were significantly (7.78-fold) higher in the PD cohort compared to the CR cohort (Fig. 3b) and this is consistent with disease progression and increase in tumour burden.

### PS co-localises with both CD138^+^ and CD138^−^ MPs in myeloma

**Fig. 4:**
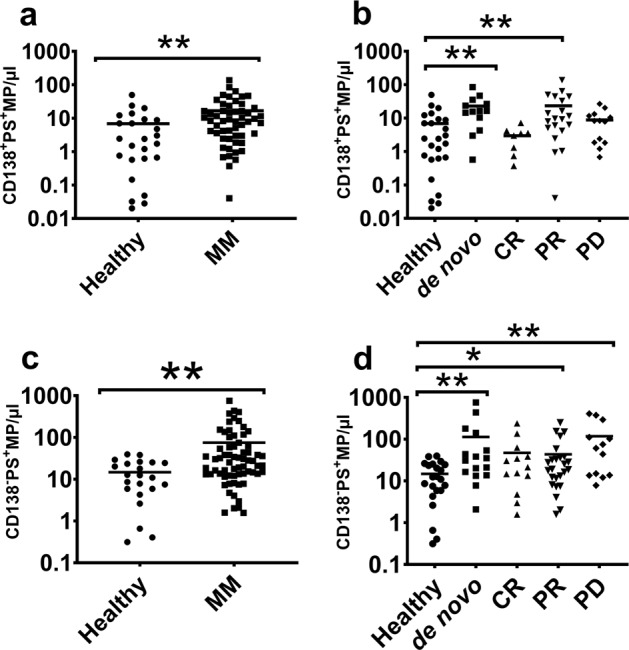
PS is enriched on CD138^−^ MPs in progressive disease. The numbers of PS^+^ MPs in context to the expression of CD138 are shown. **a** PS^**+**^MPs in the CD138^**+**^ MP subset were significantly elevated in MM patients compared to the healthy volunteers (*p* = 0.0041, *U* = 405.5). **b** CD138^**+**^PS^**+**^MP levels in the de novo and PR (*p* = 0.007, *U* = 134) cohorts were significantly higher relative to healthy volunteers, while the differences between CR or PD and healthy volunteers were not significant. **c** PS^**+**^MPs in the CD138^**−**^ MP subset were significantly elevated in MM patients relative to healthy volunteers (*p* = 0.001, *U* = 406). **d** CD138^**−**^PS^**+**^ MPs were significantly higher in de novo (*p* = 0.004, *U* = 80), PD (*p* = 0.001, *U* = 52) and PR (*p* = 0.043, *U* = 180) cohorts relative to healthy volunteers. There was no significant difference in CR cohort relative to healthy volunteers. Mann–Whitney *U* test was conducted to generate *p* values and the data are represented as mean. *p < 0.05; **p < 0.01.

Figure 4a shows the CD138^**+**^ PS^**+**^ MP counts of myeloma patients compared to healthy subjects. We observed a significant 2.4-fold increase in CD138^**+**^ PS^**+**^ MPs in the total patient cohort compared to healthy subjects. Specifically, the de novo and PR cohorts showed a 3.3- and 3.4-fold increase, respectively, in CD138^**+**^ PS^**+**^ MP. We observed no significant difference in the CR or PD patients relative to healthy subjects (Fig. 4b).

Figure 4c shows the CD138^**−**^PS^**+**^ MP counts of myeloma patients compared to healthy subjects. We observed a significant 5.1-fold increase in CD138^**−**^PS^**+**^ MPs in patients compared to healthy subjects. The de novo and PD patients had 7.6- and 8-fold greater numbers of CD138^−^ PS^**+**^ MPs, respectively, compared to healthy subjects. We also observed significantly higher numbers of CD138^−^ PS^+^ MPs (2.9-fold) in PR patients compared to healthy volunteers (Fig. 4d). We did not observe any significant difference in CD138^−^ PS^+^ MP counts across patients in CR compared to healthy subjects (Fig. 4d).

### A CD138^−^ P-gp^+^ CD34^+^ MP signature corresponds to disease progression and treatment unresponsiveness

Cancer stem cells are found in myeloma and many cancers. These ‘side populations’, as they are often referred to in flow cytometry, are characterised by: the presence of surface markers selectively expressed on cancer stem cells, which are used to isolate these cells, and the ability to extrude dyes such as Hoechst 33342. Stem cells are typically resistant to chemotherapeutics, and in the context of myeloma, are phenotypically CD138^−^^34,38–40^. Likewise, CD34 is a well-established hematopoietic stem-cell marker belonging to the CD34 family of sialomucins^41^. CD34 is also expressed on a minor subpopulation of myeloma stem-cell clones^42^.

We phenotyped for the presence of this MP signature in 11 myeloma patients during the course of treatment (Table 1A, B). Table 1A details the MP phenotype of a panel of five individual patients (patients 1–5), each representative of a distinct clinical response state. The study included patients with: (a) aggressive progressive disease, patient 1; (b) progressive disease, patient 2 (Supplementary Fig. 2A, B); (c) stable disease, patient 3 (Supplementary Fig. 2C, D); (d) partial remission, patient 4 (Supplementary Fig. 3); (e) long-term survivor in remission, patient 5. Table 1B shows the longitudinal profile of the same MP markers in seven patients (patients 4, 6–11) during the course of treatment over time.

**Table 1:** A: MP signature in MM patients representative of different clinical response states.

Patient	Response state	Total MP count (CD41a^−^)		CD41a^−^							
				CD138^−^				CD138^+^			
		P-gp+	CD34+	P-gp+ CD34+	P-gp− CD34+	P-gp+ CD34−	P-gp+ CD34+ PS+	P-gp+ CD34+	P-gp− CD34+	P-gp+ CD34−	P-gp+ CD34+ PS+
Patient 1	PD (Aggressive)	155.2	496.8	12.5	28.5	56.4	5	0.3	0.3	0	0
Patient 2	PD	60	40.5	4.78	74	60	1.1	0.5	5	3	0.4
Patient 3	Stable	6.3	5.13	4.7	18.5	23	1.6	0.2	1.2	1	0.3
Patient 4	PR	10	15.13	7.2	63	36.5	2.5	0.5	4	2.2	0.3
Patient 5	Remission (CR later)	6.3	5.13	2.5	14.4	53	0.5	3	4.5	2.4	1.17

**Table 1:** B: Longitudinal evolution of MP signature in MM patients.

Patient	Gender	Time line	Response state	CD138−				CD138+			
				P-gp+ CD34+	P-gp− CD34+	P-gp+ CD34−	P-gp+ CD34+ PS+	P-gp+ CD34+	P-gp− CD34+	P-gp+ CD34−	Pgp+ CD34+ PS+
Patient 4 (MM74)	M	14/03/2014	Diagnosis	0.3	1.63	13.31	0.03	0.1	0.81	1.97	0.03
		2/04/2014	CyBorD induction	1.83	16.1	43.1	0.03	0.13	0.88	2.64	0.03
		29/04/2014	PR	1.9	8.05	299.58	0.1	0.47	0.47	10.56	0.16
		6/05/2014	PR	7.09	14.7	322.88	0.27	0.64	0.61	18.88	0.37
		10/06/2014	PR	5.12	27.07	387.52	1.32	1.66	2.3	20.14	1.42
		17/06/2014	PR	4.61	27.1	258.82	1.15	1.73	4.48	37.39	1.35
		30/06/2014	PR	1.76	10.69	49.65	1.52	2.34	5.23	9.61	1.69
		21/07/2014	PR	1.18	2.71	31.45	0.23	0.5	1.18	6.24	0.33
Patient 6 (MM34)	F	29/11/2013	PD (terminal)	122	775.32	238.44	3.46	4.51	4.92	3.6	3.66
		13/12/2013	PD	152.17	788.73	208.99	7.91	9.13	17.59	4.6	8.35
		17/01/2014	PD	77.57	312.01	69.32	5.97	6.89	13.65	4.5	6.08
		20/02/2014	PD	61.68	130.66	207.84	6.89	7.81	5.36	12.73	6.99
		11/03/2014	PD	98.8	485.65	150.64	6.55	9.17	10.25	8.79	6.79
Patient 7 (MM19)	M	30/09/2013	Stable	38.17	177.54	43.4	5.06	6.24	8.79	10.12	5.26
		4/11/2013	PD	27.78	223.7	58.04	3.49	4.1	6.48	8.76	3.63
Patient 8 (MM79)	M	1/05/2014	PR	4.95	9.57	22.38	0.1	0.16	0.3	0.98	0.1
		10/06/2014	PR	6.99	45.85	37.7	0.5	0.78	3.53	2.68	0.54
		17/06/2014	PR	8.59	40.28	119.42	0.4	0.4	0.57	2.85	0.4
		7/07/2014	PR	10.83	145.51	74.65	1.01	1.42	6.38	6.99	1.05
Patient 9 (MM49)	M	3/03/2014	PR	4.27	16.5	31.55	0.4	0.61	2.13	2.27	0.47
		5/05/2014	PD (suspecting)	6.18	15.55	21.43	1.15	1.56	2.41	1.35	1.32
Patient 10 (MM41)	F	26/08/2013	PR	2.13	2.24	185.55	0.13	0.61	0.2	14.26	0.2
		25/09/2013	ASCT	76.32	0.57	7.5	0.37	0.61	0.23	7.26	0.3
		20/11/2013	PR	3.05	8.08	283.65	0.3	1.35	1.01	7.88	0.54
		5/12/2013	PR	27.47	36.03	2861.08	3.43	4.07	0.84	70.2	3.43
		14/02/2014	PR	6.08	11.2	721.72	0.37	5.12	0.61	48.67	0
		14/03/2014	PR	2.71	2.37	205.6	0.74	1.22	0.23	15.35	0.98
		4/04/2014	PR	1.32	2.24	237.25	0.16	0.98	0.3	15.52	0.27
		13/05/2014	PR	4.48	3.56	269.32	0.74	1.42	0.27	34.03	0.98
		10/06/2014	PR	0.44	1.18	216.02	0.2	0.57	0.33	12.49	0.33
		10/07/2014	PR	3.83	7.23	185.11	0.44	1.01	0.91	9.2	0.54
		3/10/2015	PR	0.78	2.41	212.29	0.78	1.22	0.5	36.14	0.81
Patient 11 (MM71)	F	24/03/2014	De novo	47.04	32.84	586.29	1.05	1.52	0.27	11.07	1.25
		24/04/2014	PR	2.92	5.36	118.71	0	0.03	0.03	0.95	0
		23/05/2014	PR	3.1	8.01	25.16	0.13	0.23	0.78	2.75	0.13

Values are expressed as MP/μl.

*MP* microparticle, *PD* progressive disease, *PR* partial remission, *CR* complete remission.

We observed that the CD138^**−**^ P-gp^**+**^ CD34^**+**^ dual-positive MP subpopulation was elevated in patients with advanced aggressive/unresponsive/terminal disease (i.e., patients 6 and 1) relative to patients in remission or those responsive to therapy (i.e., patients 4, 5, 8, 9, 10, 11). In the following sections, we provide details with respect to patients 1 and 5 for simplicity, and which represent two clearly distinct clinical states. A more detailed patient history of these and the other patients examined as part of the longitudinal study are detailed in Supplementary Materials 1, 2.

#### P-gp^+^ MP numbers in a 58-year-old female patient with aggressive disease (patient 1)

**Fig. 5:**
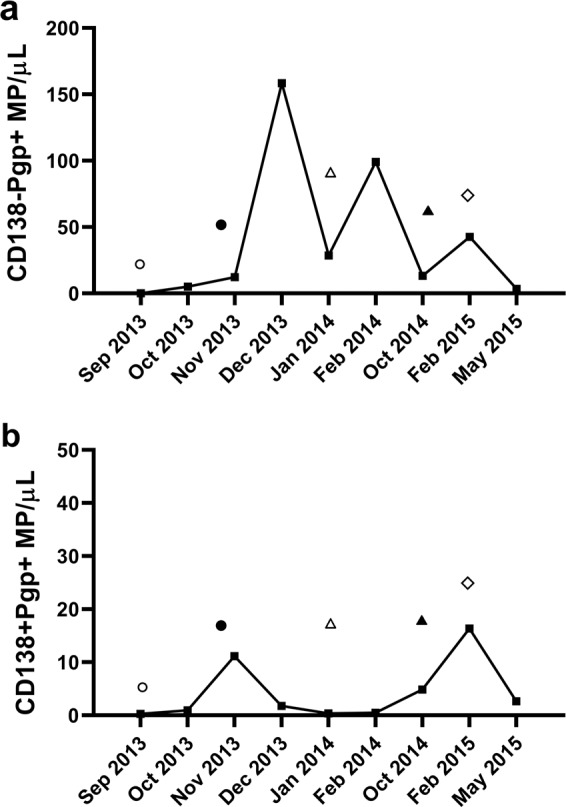
P-gp^+^MPs in a 58-year-old female patient with aggressive disease (patient 1). The P-gp^**+**^ MP counts (*Y*-axis) and time of MP sampling post diagnosis (*X*-axis) are shown. **a** CD138^**−**^P-gp^**+**^ MP numbers during treatment (CyBorD, circle; BorD (Cyclophosphamide discontinued), filled circle; VTD, triangle; lenalidomide/dexamethasone, filled triangle; D-PACE and melphalan, diamond). **b** CD138^**+**^ P-gp^**+**^ MP numbers during treatment.

Figure 5 represents the levels of different P-gp^**+**^ MP subpopulations of patient 1 in response to treatment. The patient’s total MPs were regularly phenotyped from the day of diagnosis in September 2013. An initial increase in P-gp^+^ MPs counts was evident 3 months following the start of treatment (December 2013), and this was elevated in the CD138^−^ subpopulation (Fig. 5a). At that time, the patient was in partial remission with a corresponding 46% decrease in bone marrow plasmacytosis from the initial 86% at diagnosis. A slight drop in the CD138^**−**^Pgp^**+**^ MPs was observed after the start of Thalidomide treatment in January 2014 (Fig. 5a). CD138^**−**^Pgp^**+**^ MPs levels peaked again in February 2014, although the patient was still in partial remission with again a further reduction of bone marrow plasmacytosis of 23% in April 2014 and a stem-cell transplantation was scheduled. However, the patient was found to have relapsed by May−June 2014 consistent with the increase in paraprotein. Consequently, we observed an increase of the levels of CD138^**−**^Pgp^**+**^ MPs prior to the clinical manifestation of relapse evidencing the capacity of the signature to detect the transition between remission and relapse before the existing clinical test used.

Following relapse, the treatment regimen was changed to lenalidomide/dexamethasone from July until October 2014. The patient further relapsed in February 2015 with a chest wall plasmacytoma, and the bone marrow plasmacytosis was reported at 60%. This relapse correlated with another increase in CD138^**−**^Pgp^**+**^ MPs. The addition of D-PACE and melphalan resulted in a partial response in May 2015 and the patient was finally given ASCT on 17 July 2015. The partial response obtained in May 2015 correlated with a decrease in CD138^**−**^Pgp^**+**^ MPs. The transplant proved to be only transiently effective and the patient presented with complicated physical manifestations in October−November 2015, became unresponsive to therapy, passing away in December 2015.

**Fig. 6:**
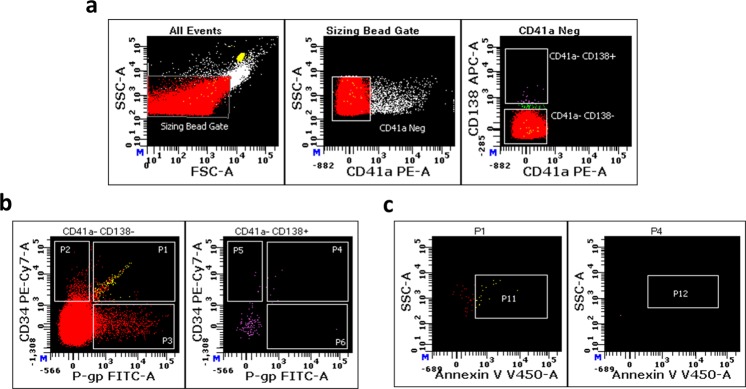
Increased ‘dual-positive’ MPs in aggressive disease (patient 1). P-gp^+^ and CD34^+^ MP counts in CD138^**−**^ and CD138^**+**^ MP populations were determined using flow cytometry. **a** A sequential gating strategy using MP size (left panel) followed by CD41a expression (middle panel) and CD138 expression (right panel) was applied to the total MP population (left panel). The total MP population (CD41a^−^) was defined based on ± staining for anti-CD41a (middle panel). **b** The total MP population was gated based on CD138 expression. We phenotyped for the presence of CD138^**−**^ P-gp^**+**^ CD34^**+**^ MPs (left panel, gate P1) and CD138^**+**^ P-gp^**+**^ CD34^**+**^ MPs (right panel, gate P4). **c** The CD138^−^ P-gp^+^ CD34^+^ and CD138^+^ P-gp^+^ CD34^+^ MP subpopulations (gates P1 and P4 of left and right panel in **b**, respectively) were gated and phenotyped for PS^+^ events using annexin V (left panel, gate P11; right panel, gate P12), respectively.

The blood sample taken in February 2015, during PD and prior to stem-cell transplantation, was also analysed for CD34 and PS expression. Figure 6 shows the flow cytometry cytograms including gating strategy used (Fig. 6a). Figure 6b shows the presence of P-gp^**+**^, CD34^**+**^ and P-gp^**+**^CD34^**+**^ MPs, particularly evident in the CD138^**−**^ population. Total CD34^**+**^ (496.81/µl) and P-gp^**+**^ (155.29/µl) MP counts were higher relative to when the patient was in PR in May 2015 (7.33/µl and 6.31/µl for CD34^**+**^ and P-gp^**+**^, respectively, data not shown).

We compared the levels of CD34^**+**^ P-gp^**+**^ MPs on CD138^**+**^ (Fig. 6b, right panel) and CD138^**−**^ (Fig. 6b, left panel) MP subpopulations. Figure 6b shows the strong presence of the dual-positive CD138^−^P-gp^**+**^ CD34^**+**^ (Fig. 6b, left panel, gate P1, 12.48/µl) in this unresponsive patient. In comparison, we detected small numbers of CD138^**+**^ P-gp^**+**^ CD34^**+**^ MPs (Fig. 6b, right panel, gate P4, 0.30/µl). We also identified additional MP subsets which were CD138^**−**^ P-gp^**+**^CD34^**−**^ (Fig. 6b, left panel, gate P3, 56.45/µl) and CD138^−^ P-gp^**−**^ CD34^**+**^ (Fig. 6b, right panel, gate P2, 28.5/µl). We did not detect MPs within the CD138^**+**^ population that were solely CD34^+^ or P-gp^**+**^ (Fig. 6b, right panel, gates P5 and P6).

The CD138^−^ and CD138^+^ dual-positive MP subpopulations were subsequently gated and phenotyped for the presence of PS using Annexin V. We detected PS^**+**^ MPs within the CD138^**−**^ P-gp^**+**^CD34^**+**^ MP population (Fig. 6c, left panel, gate P11, 5/µl). We did not detect Annexin V-positive events on CD138^**+**^ P-gp^**+**^CD34^**+**^ MPs (Fig. 6c, right panel, gate P12).

#### 62-year-old male patient in remission—long-term survivor (patient 5)

**Fig. 7:**
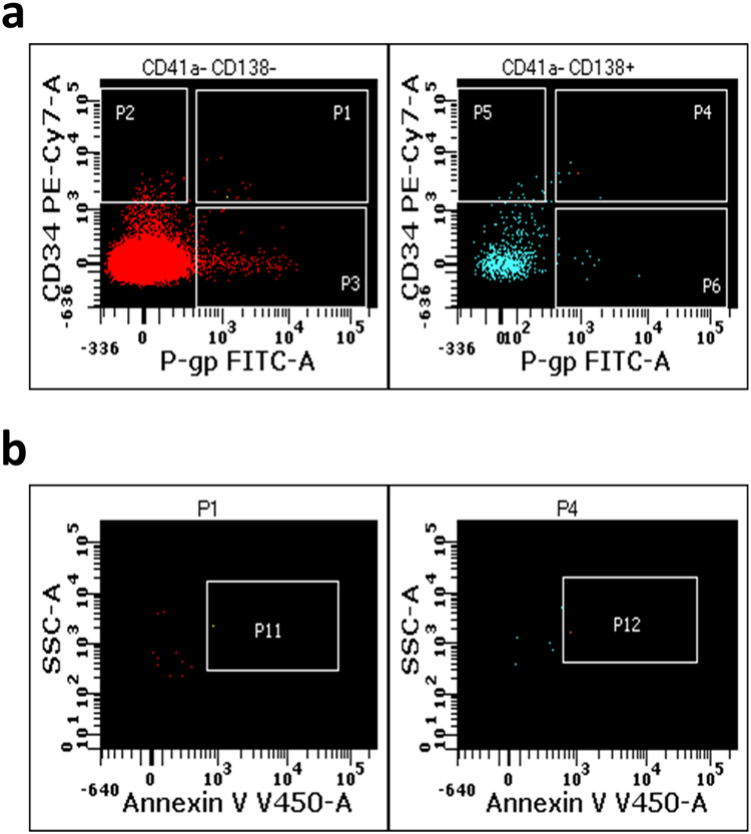
62-year-old male patient in remission—long-term survivor (patient 5). The presence of P-gp^**+**^ and CD34^**+**^ MPs in CD138^−^ (left panel) and CD138^**+**^ (right panel) MP populations was determined by flow cytometry for patient 5. **a** The total MP population was gated based on CD138 expression (left panel, CD138^−^; right panel, CD138^+^, respectively). We phenotyped for CD138^−^ P-gp^**+**^ CD34^**+**^ MPs (left panel, gate P1) and CD138^**+**^ P-gp^**+**^ CD34^**+**^ MPs (right panel, gate P4). **b** The CD138 MP subpopulations (gates P1 and P4 of left and right panel, respectively) were also gated and phenotyped for the presence of PS using annexin V (left panel, gate P11, events; right panel, gate P12), respectively.

At the time of sampling on 5 May 2015, patient 5 was responding well and eventually achieved stringent complete remission with ongoing chemotherapy. This patient is a long-term survivor (>10 years) with successful therapeutic interventions. A flow cytometric scatter plot depicting the different CD138^+/^^−^ subpopulations for this patient is shown in Fig. 7a, b. At the time of sampling we detected lower CD34^**+**^ (5.13/µl) and P-gp^**+**^ MPs (6.3/µl) numbers within the total MP population, in patient 5, compared to patient 1. We also detected a consistently lower number of dual-positive (CD138^**−**^ P-gp^**+**^ CD34^**+**^**)** MPs (Fig. 7a, left panel, gate P1, 2.54/µl) and a CD138^**+**^ P-gp^**+**^CD34^**+**^ MP population (Fig. 7a, right panel gate P4, 3.0/µl). We detected a subset of CD138^**−**^ P-gp^**+**^CD34^−^ MPs (Fig. 7a, left panel, gate P3, 52.83/µl) and CD138^**−**^ P-gp^**−**^ CD34^**+**^ MPs (Fig. 7a, left panel, gate P2, 14.46/µl). We also observed a small subpopulation of CD138^**+**^ P-gp^**−**^ CD34^**+**^ MPs (Fig. 7a, right panel, gate P5, 4.5/µl) and CD138^**+**^ P-gp^**+**^ CD34^−^ MPs (Fig. 7a, right panel, gate P6, 2.4/µl).

The CD138 MP subpopulations were gated, phenotyped and quantified for PS^+^ MPs. We detected a lower number of CD138^**−**^ P-gp^**+**^CD34^**+**^ PS^**+**^ MPs (Fig. 7b, left panel, gate P11, 0.5/µl) and CD138^**+**^ P-gp^+^ CD34^+^ PS^**+**^ MPs (Fig. 7b, right panel, gate P12, 1.17/ µl), compared to patient 1.

## Discussion

We describe a liquid biopsy that monitors for the presence of an MDR biomarker ‘signature’. This minimally invasive test accounts for tumour heterogeneity characteristic of multisite tumour infiltrates, can test for the presence of MDR during routine follow-up and allows for simultaneous analysis of disease burden. The test complements existing gold standard tests, has potential to support clinical staging criteria and streamlines easily into existing hospital workflows.

We demonstrate for the first time the presence of circulating MP subpopulations in the context of P-gp expression in myeloma patients. We show that patients have higher numbers of P-gp^**+**^ MPs compared to healthy subjects, and this is associated with poor therapeutic response. Our earlier work showed that MPs shed from MDR cancer cells carry functional P-gp from the cell of origin^26,27^. P-gp expression is typically induced in malignant cells following drug exposure; however, it can also be inherently expressed. This is consistent with our observation that 35% of the de novo patients had elevated P-gp^**+**^ MP levels. Likewise, elevated P-gp^**+**^ MP levels were also observed in patients with PD following treatment.

CD138 is the most useful marker for plasma cells and it is the most appropriate when a single marker is used^43^. However, we observed that P-gp^**+**^ MPs were predominantly CD138^**−**^. Within the total MP population, we identified a number of different subpopulations based on the expression of CD138, P-gp, CD34 and PS. Amongst these, the presence of a ‘dual-positive’ (CD138^**−**^CD34^**+**^P-gp^**+**^) population appears to be associated with an unresponsive state.

The predominant expression of the aforementioned markers in the CD138^−^ subset of MPs may be a consequence of CD138 shedding, which has been observed in aggressive disease^34^.

Another possibility is the emergence of a ‘side population’ composed of putative myeloma stem cells during disease progression. These cells are typically CD138^−^ and express high levels of functional P-gp^44–46^. Lower expression of CD138 on plasma cells is indicative of an immature phenotype, poor prognosis and lower sensitivity to lenalidomide treatment^38–40^.

The longitudinal data shown in Table 1B confirm the predominance of CD138^**−**^ subtypes in accordance with clinical response states. For example, patient 6 (Supplementary Fig. 4) is an MM patient with terminal disease. Patient 6 underwent many different treatment regimens and became treatment unresponsive. We observed elevated counts of the ‘dual-positive’ (CD138^**−**^ P-gp^+^CD34^+^) MPs in this patient, which were higher relative to treatment-responsive patients, further validating our observations.

PS is an ubiquitous MP marker arising from the loss of phospholipid asymmetry during MP biogenesis^47^. However, PS is not an absolute MP marker and its expression is variable in MP populations^48,49^. Nevertheless, PS is emerging as an important mediator in extracellular vesicle biology and as a target in anticancer therapy. Its cell surface presence was shown to enable cancer cell evasion from physiological immune checkpoints^50^. Furthermore, a recent study suggested a pro-angiogenic role for PS exposed on MPs surface^37^. PS is also known to be a highly immunosuppressive phospholipid^51^. Interestingly, the exposure of PS on MPs was shown to contribute to hypercoagulable states^52^, and higher rates of thromboembolic events have been associated with the use of oral immunomodulatory drugs (IMiDs), thalidomide in particular^53^. We observed significantly elevated PS^**+**^ MPs across all disease stages except in CR, suggesting that their levels may be associated with ‘active disease’ states. This hypothesis is further corroborated by the significantly higher PS^+^ levels we observed in PD compared to CR. We also observed significantly greater numbers of PS^+^ MPs among the CD138^**−**^ MP population in some patients (specifically, de novo and PD). In PD patients, the levels of CD138^**−**^ PS^**+**^ MPs may also be an indicator of tumour burden.

Unlike P-gp, there was also a significantly greater number of PS^+^ MPs in the CD138^**+**^ MP subpopulation in MM patients relative to healthy subjects (specifically for the de novo and PR cohort). PS^**+**^ events in the ‘dual-positive’ MP population of the five patients examined were in the following order: aggressive PD > PR > stable > PD > remission patients (Table 1A). The significance of the increased PS^**+**^ MP numbers in myeloma is currently unknown and may be linked to the dissemination of malignant cells to extramedullary sites during disease progression^37^.

This study, together with our earlier work^23^, identifies many MP subtypes in myeloma patients. CD138^**+**^ MPs provide a marker of plasma-cell burden, while the presence of the ‘dual-positive’ MPs (CD138^**−**^CD34^**+**^P-gp^**+**^) of ‘stem-cell-like’ origin appear to be a marker of disease progression and treatment unresponsiveness in patients, specifically with aggressive disease.

CD138 cannot be considered a ‘static’ biomarker of MM disease evolution as the presence of a CD138^−^ population increases in aggressive disease. This has important implications in how we define the utility of biomarkers at each stage of disease.

In conclusion, MPs provide a surrogate marker of their cells of origin, which in the case of myeloma are predominantly confined to the bone marrow. We provide evidence that MDR in patients with MM can be detected and serially monitored by analysing MPs in blood samples in the context of a ‘liquid biopsy’. Our results indicate the presence of markers of MDR on MPs of stem-cell-like origin. Stem cells are a reservoir of P-gp-positive cells, the levels of which appear to correspond to disease progression. This has significant implications in the design of effective treatment strategies, including targeted approaches against distinct cell clones with discrete phenotypes. The shifting dominance of these signatures present at various times must be considered during the design of treatment interventions.

The work we present here depicts a personalised approach with prognostic potential in determining the presence of MDR in MM, whereby the development of MDR can be serially and minimally invasively monitored by analysing circulating MPs in the context of a liquid biopsy. This work, besides introducing new exciting insights into the molecular mechanisms contributing to MDR and treatment failure in MM, demonstrates potential as a new clinical test to complement existing procedures used for the management of myeloma.

## Supplementary information

Supplementary Figure 1

Supplementary Figure 2

Supplementary Figure 3

Supplementary Figure 4

Legends for Supplementary Figures

Supplementary Material 1 - Patients 1 to 5 Clinical History

Supplementary Material 2 - Longitudinal Patients Clinical History

Supplementary Table 1 - Reagents specifications and details

Supplementary Table 2 - BD Fortessa FCM Configuration

Supplementary Table 3 - BD LSRII FCM Configuration

## Data Availability

The data used to support the findings of this study are available from the corresponding author upon request.
