# Supplementary figures and images for: A liquid biopsy to detect multidrug resistance and disease burden in multiple myeloma
Source: Blood Cancer J. 2020 Mar 13;10(3):37. doi: 10.1038/s41408-020-0304-7 (PMC7070076; doi:10.1038/s41408-020-0304-7)

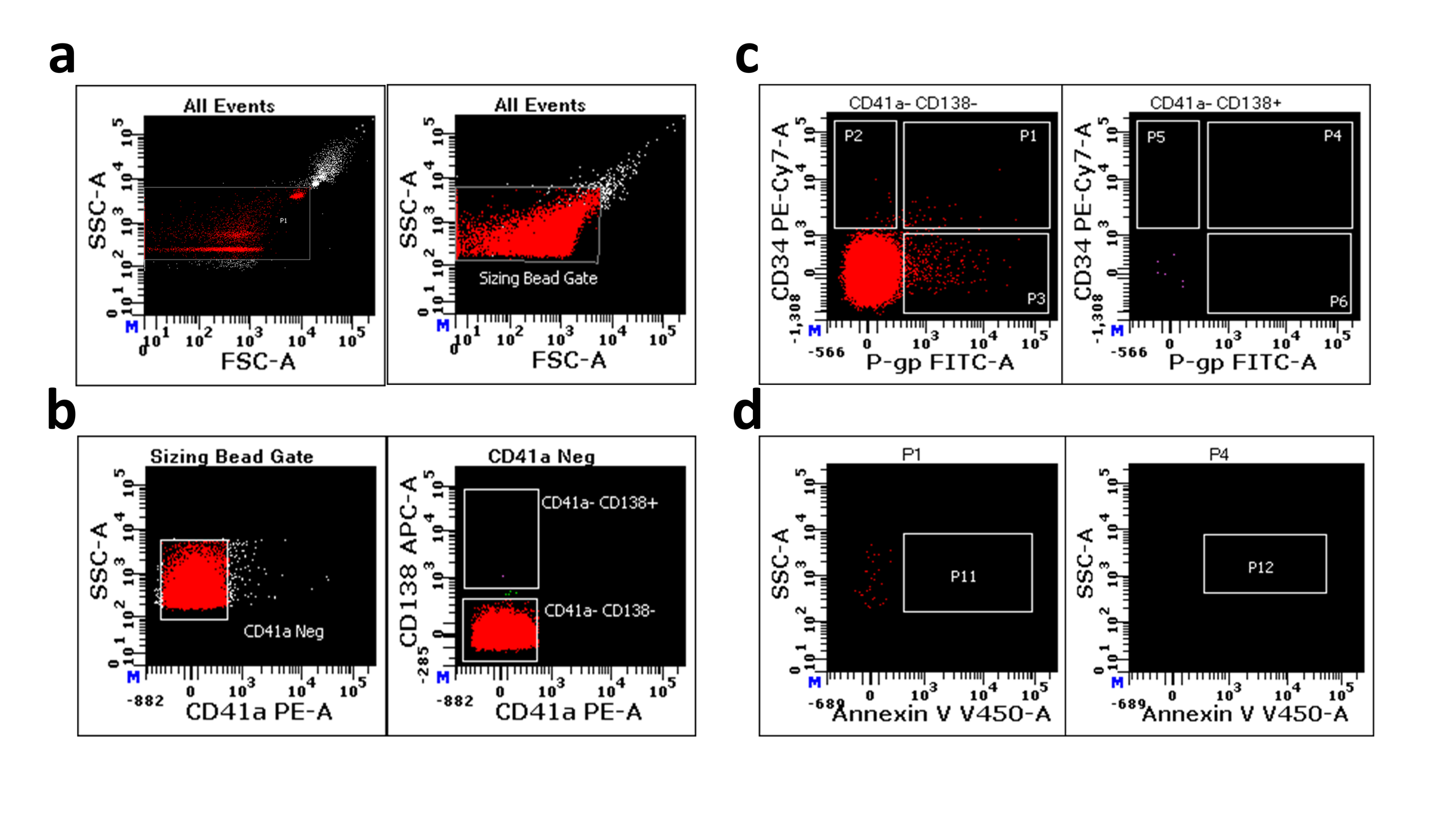

Supplement: Supplementary file 1 — Supplementary Figure 1 [file 41408_2020_304_MOESM1_ESM.tif]

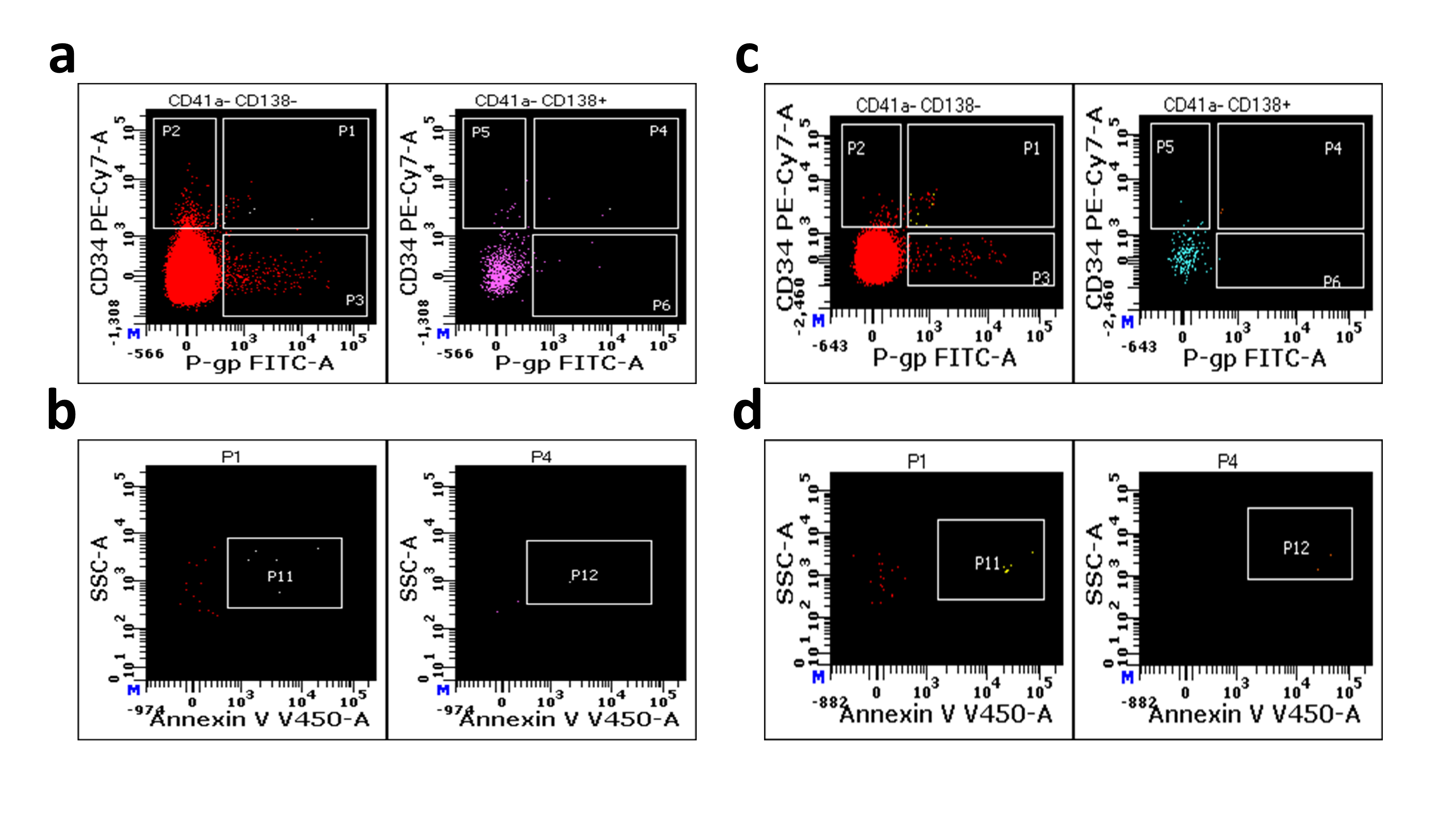

Supplement: Supplementary file 2 — Supplementary Figure 2 [file 41408_2020_304_MOESM2_ESM.tif]

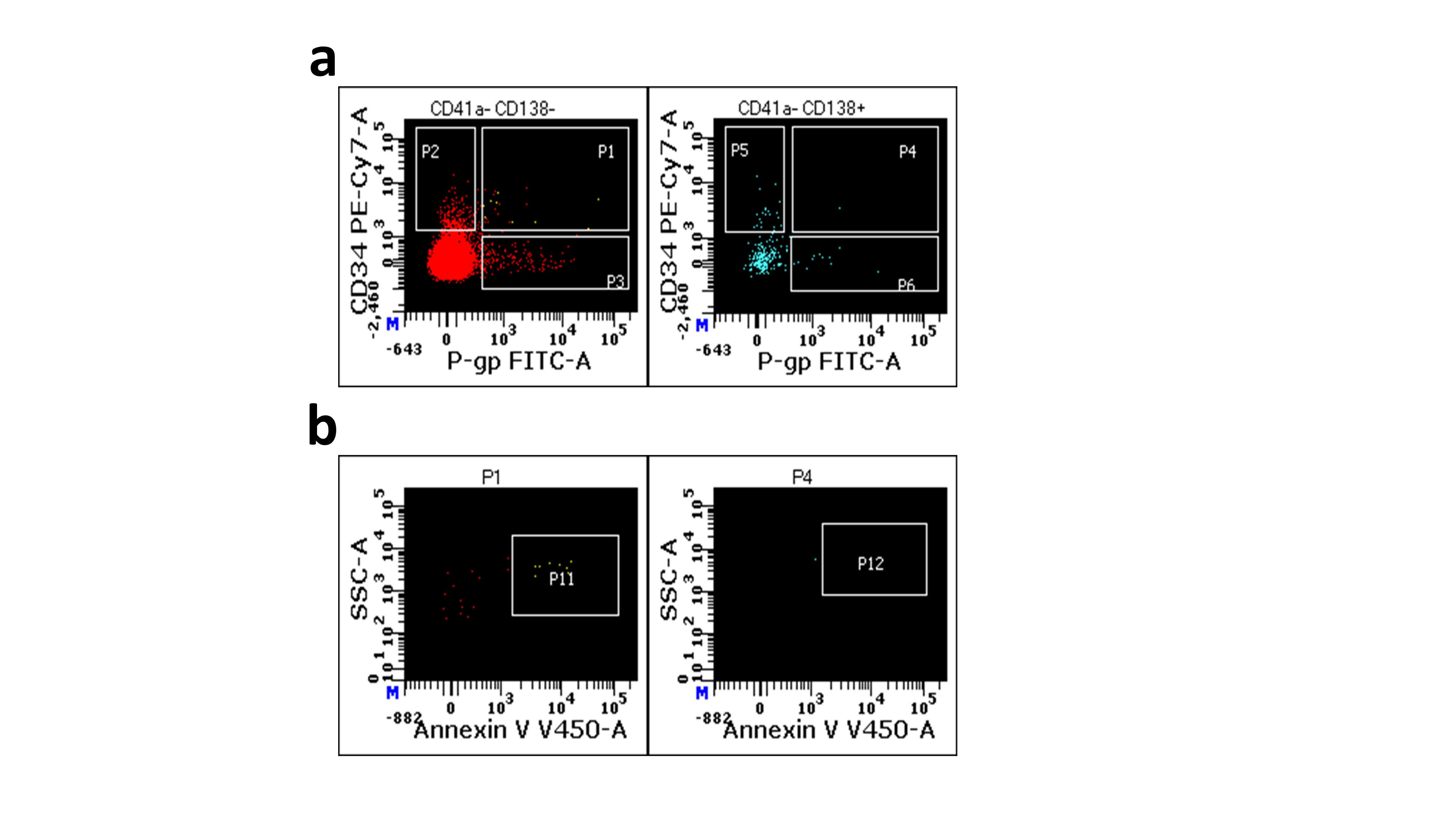

Supplement: Supplementary file 3 — Supplementary Figure 3 [file 41408_2020_304_MOESM3_ESM.tif]

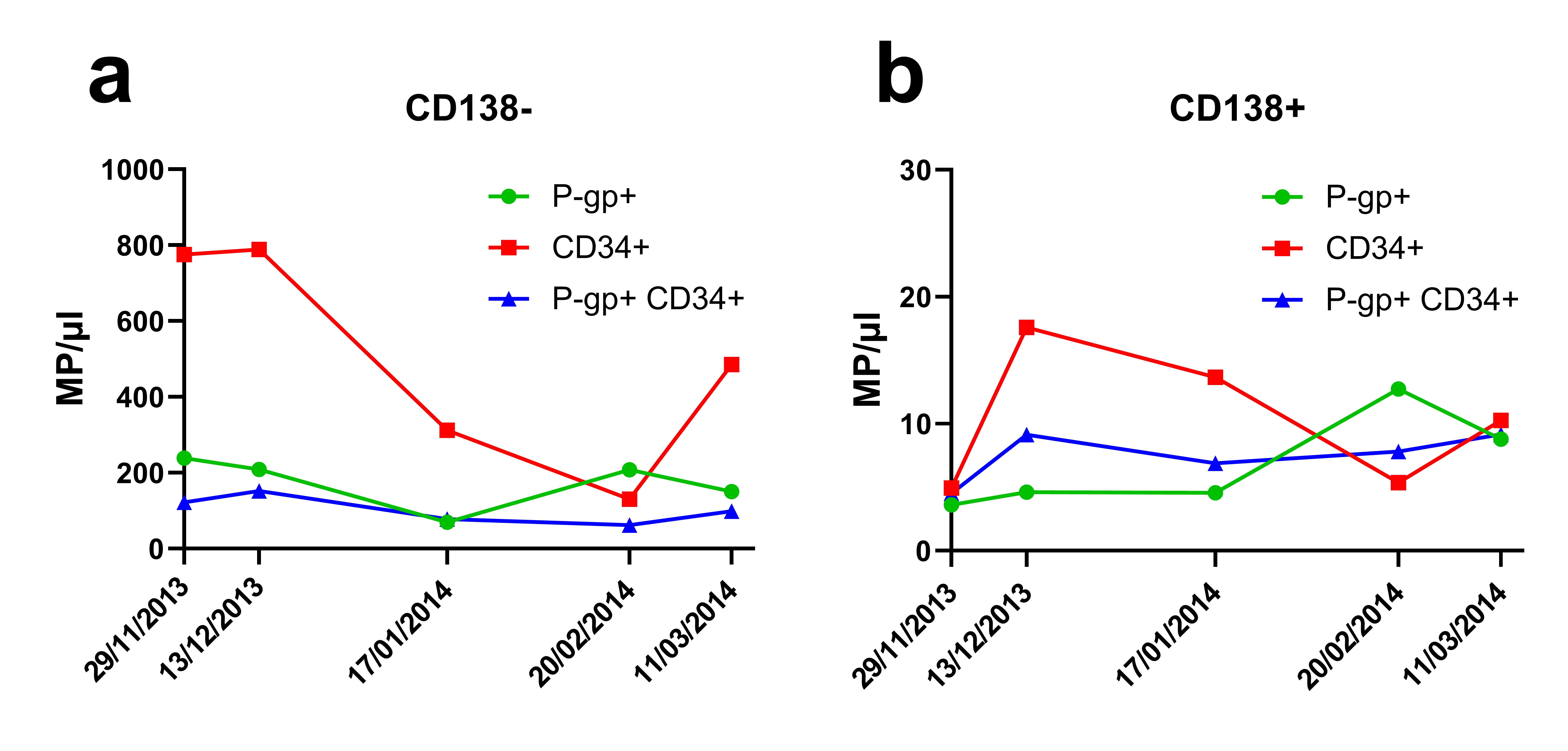

Supplement: Supplementary file 4 — Supplementary Figure 4 [file 41408_2020_304_MOESM4_ESM.tif]
