# Supplementary material for: A liquid biopsy to detect multidrug resistance and disease burden in multiple myeloma
Source: Blood Cancer J. 2020 Mar 13;10(3):37. doi: 10.1038/s41408-020-0304-7 (PMC7070076; doi:10.1038/s41408-020-0304-7)
Supplement: Supplementary file 5 — Legends for Supplementary Figures [file 41408_2020_304_MOESM5_ESM.docx]

**Supplementary Figures Legends**

**Supplementary Figure 1 - Gating strategy to define parameters for +/- staining**

(A) Latex beads of known diameter (0.3 – 1.1 µm) were resuspended in PBS and analysed using the LSR Fortessa X20. MP size gates were defined as in left panel. The MP size gate was applied to the patients MP population (right panel). **(B)** A sequential gating strategy using MP size gate followed by gating for CD41a (left panel) and CD138 (right panel) was applied to the patients MPs. **(C)** CD138^-^ (left panel) and CD138^+^ (right panel) populations were gated for P-gp and CD34 positivity **(D)** CD138^-^Pgp^+^CD34^+^ (P1, left panel) and CD138^+^Pgp^+^CD34^+^ (P4, right panel) were finally gated for PS expression with Annexin V.

**Supplementary Figure 2 - 66-year-old female patient with progressive disease (Patient 2) and 63-year- old male (Patient 3) in a stable condition.**

The presence of P-gp**^+^** and CD34**^+^** MPs in CD138**^-^** (red events) and CD138**^+^** (blue events) subpopulations was determined by flow cytometry for patient 2 (A, B) and 3 (C, D)

**(A)** The total MP population was gated based on CD138 expression (CD138^-^: left panel, red events; CD138^+^: right panel, blue events, respectively). We phenotyped for CD138**^-^** P-gp**^+^** CD34**^+^** MPs (left panel, gate P1) and CD138**^+^** P-gp**^+^** CD34**^+^** MPs (right panel, gate P4)**^.^ (B)** The CD138 MP subpopulations (gate P1 & P4 of left and right panel respectively) were also gated and phenotyped for the presence PS using annexin V (left panel, gate P11, yellow events; right panel, gate P12, orange events respectively). **(C)** and **(D)** represent the same gating respectively for patient 3.

**Supplementary Figure 3 - 71-year-old male patient in partial remission (Patient 4)**

The presence of P-gp**^+^** and CD34**^+^** MPs in CD138**^-^** and CD138**^+^** subpopulations was determined by flow cytometry for patient 4 **(A)** The total MP population was gated based on CD138 expression (left panel, red events, right panel, blue events, respectively). We phenotyped for CD138**^-^** P-gp**^+^** CD34**^+^** MPs (left panel, gate P1) and CD138**^+^** P-gp**^+^** CD34**^+^** MPs (right panel, gate P4)**^.^ (B)** The CD138 MP subpopulations in (A) were also gated and phenotyped for the presence PS using annexin V (left panel, gate P11, yellow events, right panel, gate P12, orange events).

**Supplementary Figure 4 –** **MP subpopulations in a terminal patient (Patient 6)**

**(A)**, CD138^-^ sub-populations (P-gp^+^ CD34^+^, P-gp^+^, CD34^+^) **(B)**, CD138^+^ sub-types (P-gp^+^ CD34^+^, P-gp, CD34^+^)
