# Supplementary material for: A liquid biopsy to detect multidrug resistance and disease burden in multiple myeloma
Source: Blood Cancer J. 2020 Mar 13;10(3):37. doi: 10.1038/s41408-020-0304-7 (PMC7070076; doi:10.1038/s41408-020-0304-7)
Supplement: Supplementary file 6 — Supplementary Material 1 - Patients 1 to 5 Clinical History [file 41408_2020_304_MOESM6_ESM.docx]

**Supplementary material 1 – Patients 1-5 Clinical History (from Table 1A)**

**Patient 1: 58-year-old female with aggressive disease.**

Patient 1 is a 58-year-old woman who was diagnosed with IgG myeloma in Sept. 2013 and presented with 86% plasmacytosis in the bone marrow aspirate. Her CD138^-^Pgp^+^ and CD138^+^Pgp^+^ MPs subpopulation levels are shown in Fig. 5 in the main text. At diagnosis, the patient’s P-gp**^+^** MP numbers were low. Induction therapy with CyBorD commenced in Sept. 2013 (circle) but in Nov. 2013 cyclophosphamide was withdrawn due to severe anaemia (filled circle). A bone marrow biopsy in Dec. 2013 showed partial response with 46% plasmacytosis. During this time, the patient’s P-gp**^+^** MP counts started to increase steadily, consistent with future development of treatment unresponsiveness and the emergence of MDR. Thalidomide was included from Jan-Apr 2014 (triangle). A follow up biopsy showed 23% reduction in plasmacytosis in Apr 2014. Paraprotein levels increased to 38.3 g/l indicating progressive disease in Jun-Jul 2014 and the treatment regimen was changed to lenalidomide/dexamethasone from Jul-Oct 2014 (filled triangle). The patient relapsed with a right side posterior mass along the chest wall in early Feb 2015 (60% plasmacytosis) while the M-protein level remained at 18g/l suggesting a transition to non-secretory myeloma (data not shown). At this time, CD138^-^P-gp^+^ MP counts increased. D-PACE and melphalan were added to the treatment regimen (diamond) and the patient achieved partial remission (~ day 495). The patient had a successful autologous stem cell transplant in Jul 2015, however, soon relapsed and became totally unreponsive to therapy in Nov 2015. The patient passed away in Dec 2015.

**Patient 2: 66-year-old female with progressive disease (PD)**

A 66-year-old female patient was diagnosed with kappa light chain myeloma in 2014. She was enrolled and treated as part of a clinical trial (MLN9708 -cyclophosphamide/dexamethasone) from Dec 2014 until Mar 2015. This was stopped in Feb 2015 due to progressive disease and a rise in kappa light chains. At the time of sampling on May 2015, she was on CyBorD therapy. During this time we observed increased CD34**^+^** (40.5/µl) and P-gp**^+^** MP numbers (60/µl) in the total MP population (Table 1A). We detected a CD138**^-^** P-gp**^+^** CD34**^+^** MP population (sup. fig, 2A, left panel, gate P1, 4.6/µl) and a CD138**^+^** P-gp**^+^** CD34**^+^** MP population **(**sup. fig. 2A , right panel, gate P4, 0.5/µl). We also detected a sub-set of CD138**^-^** P-gp**^+^** CD34^-^ MPs (sup. fig. 2A , left panel, gate P3, 58.8/µl) and CD138**^+^** P-gp**^-^** CD34**^+^** MPs (sup. fig. 2A , right panel**,** gate P6, 3/µl).

The CD138 MP dual sub-populations were gated and phenotyped and quantified for PS^+^ events using annexin V. We detected a small presence of CD138^-^ P-gp**^+^**CD34**^+^** PS**^+^** MPs **(**sup. fig. 2B, left panel, gate P11, 1.1/µl) in this patient. CD138**^+^**P-gp**^+^**CD34**^+^** PS**^+^** MP levels were also low (sup fig.2B, right panel, gate P12, 0.4/µl) (Table 1A).

In comparison to patient 1 with aggressive disease, patient 2 with progressive disease demonstrated lower levels of the ‘dual positive’ population and showed lower PS^+^ MPs.

**Patient 3: 63-year-old male in stable condition**

A 63-year-old male with stable disease at the time of sampling was diagnosed with IgG kappa MM in 2011 (smoldering myeloma 2008, active myeloma July 2011). Induction therapy consisted of 6 cycles of cyclophosphamide, thalidomide and dexamethasone followed by autologous stem cell transplant on 30th Mar 2012. The patient experienced severe peripheral neuropathy associated with thalidomide and an increase in serum paraprotein levels, which resulted in a change to lenalidomide and dexamethasone in Jul 2012. At the time of sampling in May 2015, the patient was on lenalidomide, dexamethasone, zometa and aspirin.

The patient presented with CD34**^+^** (5.13/µl) and P-gp**^+^** (6.3/µl) MPs in the total MP population (Table 1A). Within this population, we detected a CD138**^-^** P-gp**^+^** CD34**^+^** MP sub**^-^**population (Sup.fig 2C, left panel, gate P1, 4.7/µl) and a CD138**^+^**P-gp**^+^**CD34**^+^** M sub-population (sup.fig 2C, right panel, gate P4, 0.2/µl). We also identified CD138**^-^**P-gp**^+^**CD34**^-^** MPs (sup.fig 2C , left panel, gate P3 23.13/µl) and CD138^-^P-gp**^-^**CD34**^+^** MPs (sup.fig 2C **,** left panel, gate P2, 18.54/µl). We also detected subpopulations of CD138**^+^**P-gp**^+^** CD34**^+^** MPs (sup.fig 2C , right panel, gate P5, 1.2/µl) CD138**^+^** P-gp**^+^** CD34**^-^** MPs (sup.fig 2C , right panel, gate P6, 1/µl).

The CD138^+/-^ dual positive subpopulations were gated, phenotyped and quantitated for PS^+^ events. CD138**^-^** P-gp**^+^**CD34**^+^** PS**^+^** MPs **(**sup.fig 2D , left panel, gate P11, 1.6/µl) and CD138**^+^** P-gp**^+^** CD34**^+^** PS**^+^** MPs (sup.fig 2D, right panel, gate P12, 0.3/µl events) (Table 1A) were not particularly elevated.

The ‘dual positive’ population was present in comparable levels to those observed for patient 2. Although we detected PS**^+^** MPs, the dual positive sub-sets were not significantly enriched with PS in comparison with patient 1 and 2.

**Patient 4: 71year old male in partial remission (PR)**

A 71-year-old male was diagnosed on Feb 2014 following a biopsy of a right shoulder mass. He presented with widely disseminated skeletal disease and multiple lesions as evidenced by positron emission tomography scan and paraprotein levels of 52.30 g/L. Induction therapy consisted of CyBorD treatment from Apr 2014. The patient achieved good partial remission (paraprotein, 5.09 g/L) after 6 cycles and treatment was stopped at 6 cycles instead of 8 due to severe peripheral neuropathy arising from bortezomib. The sample tested in Table 1A was taken in August 2014. We detected CD34**^+^** (15.13/µl) and P-gp**^+^** MPs (10/µl) in the total MP population (Table 1A). We also detected CD138**^-^** P-gp**^+^** CD34**^+^** MPs (sup.fig 3A , left panel, gate P1, 7.2/µl) and CD138**^+^** P-gp**^+^** CD34**^+^** MPs (sup.fig 3A , right panel, gate P4, 0.5/µl). We also detected a sub-set of CD138**^-^** P-gp**^+^** CD34^-^  MPs (sup.fig 3A, right panel, gate P3, 36.53/µl) and CD138**^-^** P-gp**^-^** CD34**^+^** MPs (sup.fig 3A**,** left panel, gate P2, 63.17/µl). We observed a small subpopulation of CD138**^+^** P-gp^-^CD34^+^ MPs (sup.fig 3A, right panel, gate P5, 4/µl) and CD138**^+^** P-gp^+^CD34^-^  MPs (sup.fig 3A, right panel, gate P6, 2.2/µl).

The CD138 MP dual positive subpopulations were phenotyped for the presence of PS^+^ MPs. We detected CD138^-^ P-gp**^+^**CD34**^+^** PS**^+^** MPs **(**sup.fig 3B , left panel, gate P11, 2.5/µl). We detected no CD138**^+^** P-gp**^+^**CD34**^+^** PS**^+^** MP events (sup.fig 3B , right panel, gate P12, 0 events) (Table 1A).

This patient presented with elevated albeit lower levels of the ‘dual positive’ MP population in comparison to patient 1. The sub-set was enriched with PS however this was again lower than what was measured for patient 1.

Overall, this patient had high P-gp on CD138^-^ MPs and relapsed in February 2015 despite very good response to induction therapy confirmed by BM biopsy (1%) in December 2014. The patient attained very good partial remission in 6 months (Lenalidomide Dexamethasone) and classified as complete remission in January 2016. However, the patient relapsed in July 2016 again showing very short-term remission periods.

**Patient 5: 62 year old male– A long-term survivor in remission**

Patient 5 is a 62-year-old male who was diagnosed at 50 years of age with IgG kappa MM by bone marrow biopsy, showing a 10-15% plasma cell infiltration. His induction regimen consisted of vincristine, adriamycin (doxorubicin) and dexamethasone. This was followed by an autologous stem cell transplant in 2007, after which he remained in an unmaintained complete remission for almost three years. He experienced a relapse in 2012 with a rise in serum paraprotein albeit he had no other issues. He was given thalidomide and achieved a very good partial response in early 2013 with bone marrow biopsy showing only 3% plasma cell infiltration and undetectable M-protein levels. His M protein levels started to increase in late 2014 and reached 17g/l in Oct 2014. The patient was subsequently enrolled and treated on a clinical trial (lenalidomide/dexamethasone plus or minus daratumumab) in Dec 2014. At the time of sampling the patient was responding well and eventually achieved stringent complete remission with ongoing chemotherapy. This patient is a long-term survivor with successful therapeutic interventions.
