# Supplementary material for: A liquid biopsy to detect multidrug resistance and disease burden in multiple myeloma
Source: Blood Cancer J. 2020 Mar 13;10(3):37. doi: 10.1038/s41408-020-0304-7 (PMC7070076; doi:10.1038/s41408-020-0304-7)
Supplement: Supplementary file 7 — Supplementary Material 2 - Longitudinal Patients Clinical History [file 41408_2020_304_MOESM7_ESM.docx]

**Supplementary Material 2 – Case descriptions of patients with longitudinal data (from Table 1B).**

**Patient 6** , a female MM patient at terminal stage at the time of sampling. The initial chemotherapy regimen including; Mephalan, Prednisone and Thalidomide had produced a good therapeutic response. Two years later the patient relapsed and was given the second-generation IMiD, lenalidomide with dexamethasone resulting in a partial response. The patient relapsed with this line of treatment and subsequently treatment was changed to include a proteasome inhibitor, Velcade (Bortezomib), and dexamethasone. With this, the patient demonstrated a good response although she relapsed after a year. Reintroduction of the same agents (Velcade and dexamethasone) caused a small improvement in response, and the patient became unresponsive as indicated by increased IgA levels. Cyclophosphamide was added subsequently and the patient responded. Treatment at the time of sampling consisted of PCAB+ Velcade. The patient had an M protein level of 44.0 g/L. The patient passed away soon after sampling on 4th Apr. 2014. Supplementary Figures 4A and 4B show the evolution of CD138+ and CD138- MP subpopulations for this patient.

**Patient 7** , a male patient who was stable on 30th September 2013 with an IgG level of 21.50 since July 2013. The patient was under a regimen of Thalidomide (50mg) alternating with 100 mg at night, together with Prednisone (50mg) for 7 days each month.

**Patient 8** , a 64 years old male with Kappa light chain multiple myeloma diagnosed in April 2014. Treatment includes VCAT induction followed by ASCT and Thalidomide Vs Velcade maintenance. This patient is generally fit and responsive to therapy. The investigation was prompted by an incidentally noted bone lesion. At the time of diagnosis, light chain measured 1600mg/l (01/05/14) and BM biopsy showed 17-50% plasma cells in the marrow. The patient was on VCAT since 10/06/14 (2 cycles) and was responding well. The patient had successful autologous stem cell transplantation on 12^th^ September 2014 and on Thaidomide/ Velcade maintenance from November 2014. Patient was doing well although reported experiences of tingling sensation in the toe in October 2014.

**Patient 9** , a male diagnosed with IgA kappa myeloma in 2009. The patient underwent ASCT in July 2009 and was in very good partial remission till 2013. The clinical notes suggested suspecting relapse as per 13^th^ May 2014. Paraprotein started steadily increasing in 2015.

**Patient 10** , a 52 years old female diagnosed with IgG kappa myeloma with a hyperdiploid karyotype in March 2013. She started on the VCAT study with Velcade / Cyclophosphamide / Dexamethasone induction but then developed a lytic skull lesion as well as left neck of femur lesion, both of which were subject to radiotherapy. For this reason the treatment regimen was interrupted and during the radiation treatment she was “maintained” on Thalidomide, Dexamethasone. She underwent autologous stem cell transplant in September 2013. Failure of Revlimid and PACE chemotherapy resulted in relapse in November 2014. The patient was dialysis dependent (3 times weekly) and experienced muscle cramps, neuropathy, widespread bone pain, bone lesions (Pentamidine, prednisone, Valtrex, Moxonidine, Hydralazine, Amlodipine). In 2015, treatment changed to Promalidomide (light chains reduced to 1110mg/l from 3020), Lyrica and fentanyl patch with endone, zometa infusions (monthly). However, light chains were increasing towards the end of 2015.

**Patient 11**, a 58-year-old female diagnosed with MGUS in December 2012, which transformed to Smoldering MM in March 2013 and subsequently to MM with a lytic lesion in the skull (June 2013). The patient deferred BM biopsy and sought alternative therapy (holistic immunotherapy with vincristine -1 mg, dox- 10 mg and cyclo- 300mg) in Germany in June 2013. In December 2013, her M protein measured 57.7g/l, which increased rapidly to 84.9mg/l by January 2014. By February 2014, there were more lesions and she was experiencing severe pain. Treatment started (CTD) in March 2014. She had a very good response to CTD as per the IgG measurement on 22^nd^ May 2014 (43g/l). ASCT was deferred, as the patient was Hepatitis B positive.
