# Supplementary material for: A liquid biopsy to detect multidrug resistance and disease burden in multiple myeloma
Source: Blood Cancer J. 2020 Mar 13;10(3):37. doi: 10.1038/s41408-020-0304-7 (PMC7070076; doi:10.1038/s41408-020-0304-7)
Supplement: Supplementary file 8 — Supplementary Table 1 - Reagents specifications and details [file 41408_2020_304_MOESM8_ESM.docx]

**Supplementary Table 1 – Reagents Specifications**

| **REAGENT** | **VENDOR** | **CATALOGUE N.** |
| --- | --- | --- |
| **CD138** - APC Antibody (Clone MI15) | BD Biosciences | 347193 |
| APC Isotype Control | BD Biosciences | 555751 |
| **P-gp** – FITC Antibody (Clone 17F9) | BD Biosciences | 557002 |
| FITC Isotype Control | BD Biosciences | 555742 |
| **CD41a** - PE Antibody (Clone HIP8) | BD Biosciences | 555467 |
| PE Isotype Control | BD Biosciences | 555749 |
| **CD34** – PE-Cy7 Antibody (Clone 8G12Y7) | BD Biosciences | 348791 |
| PE-Cy7 Isotype Control | BD Biosciences | 348788 |
| **AnnexinV** – V450 | BD Biosciences | 560506 |
| Annexin V Binding Buffer 10X Concentrate | BD Biosciences | 556454 |
| CompBeads Compensation Particles, Anti-Mouse Ig, k | BD Biosciences | 552843 |
| TruCount Tubes | BD Biosciences | 340334 |
| Sphero Rainbow Calibration Particles | BD Biosciences | 559123 |
| 1.1 µm Latex Beads | Sigma-Aldrich | LB11 |
| 0.3 µm Latex Beads | Sigma-Aldrich | LB3 |
