# Supplementary material for: A liquid biopsy to detect multidrug resistance and disease burden in multiple myeloma
Source: Blood Cancer J. 2020 Mar 13;10(3):37. doi: 10.1038/s41408-020-0304-7 (PMC7070076; doi:10.1038/s41408-020-0304-7)
Supplement: Supplementary file 9 — Supplementary Table 2 - BD Fortessa FCM Configuration [file 41408_2020_304_MOESM9_ESM.docx]

**Supplementary Table 2 – BD LSR Fortessa™ X-20 Lasers and Filters Configuration**

| **BD LSR Fortessa™ X-20** | | | |
| --- | --- | --- | --- |
| **Laser** | **Detector** | **Filter (BP)** | **Channel** |
| Blue, 488 nm, 100 mW | PMT1 |  | FSC |
|  | PMT2 | 488/10 | SSC |
|  | PMT3 | 530/30 | FITC |
|  | PMT4 | 575/26 | PE |
|  | PMT7 | 780/60 | PE-Cy™7 |
| Violet, 405 nm, 50 mW | PMT8 | 450/50 | V450 |
|  |  |  |  |
|  |  |  |  |
|  |  |  |  |
| Red, 635 nm,  40 mW | PMT14 | 670/30 | APC |
|  |  |  |  |
|  |  |  |  |
